# Depth of intact vascular plexus – visualized with optical coherence tomography – correlates to burn depth in thoracic thermic injuries in children

**DOI:** 10.1515/iss-2023-0066

**Published:** 2024-06-12

**Authors:** Valerie Dalicho, Tina Straube, Kathrin Kelly, Beke Larsen, Lutz Wünsch, Judith Lindert

**Affiliations:** Paediatric Burn Center Lübeck, Department of Paediatric Surgery, University Hospital Lübeck, Lübeck, Germany; Department of Paediatric Surgery, University Hospital Rostock Rostock, Germany

**Keywords:** pediatric burns, optical coherence tomography, burn depth assessment, burns thorax

## Abstract

**Objectives:**

Deep thermal injuries are among the most serious injuries in childhood, often resulting in scarring and functional impairment. However, accurate assessment of burn depth by clinical judgment is challenging. Optical coherence tomography (OCT) provides structural images of the skin and can detect blood flow within the papillary plexus. In this study, we determined the depth of the capillary network in healthy and thermally injured skin and compared it with clinical assessment.

**Methods:**

In 25 children between 7 months and 15 years of age (mean age 3.5 years (SD±4.14)) with thermal injuries of the ventral thoracic wall, we determined the depth of the capillary network using OCT. Measurements were performed on healthy skin and at the center of the thermal injury (16 grade IIa, 9 grade IIb). Comparisons were made between healthy skin and thermal injury.

**Results:**

The capillary network of the papillary plexus in healthy skin was detected at 0.33 mm (SD±0.06) from the surface. In grade IIb injuries, the depth of the capillary network was 0.36 mm (SD±0.06) and in grade IIa injuries 0.23 mm (SD±0.04) (Mann–Whitney U test: p<0.001). The overall prediction accuracy is 84 %.

**Conclusions:**

OCT can reliably detect and differentiate the depth of the capillary network in both healthy and burned skin. In clinical IIa wounds, the capillary network appears more superficial due to the loss of the epidermis, but it is still present in the upper layer, indicating a good prognosis for spontaneous healing. In clinical grade IIb wounds, the papillary plexus was visualized deeper, which is a sign of impaired blood flow.

## Introduction

Deep thermal injuries are among the most serious childhood injuries, with scarring and skin textural changes leading to cosmetic and functional impairment [1, 2]. The severity of thermal injuries depends on their size, depth, and location [1]. A burn that does not heal within 14 days is likely to produce a permanent scar [3, 4].

While grade I and grade III injuries are easy to recognize, intermediate (grade II) injuries are more challenging to assess correctly. Poor judgment can lead to over- or undertreatment with unsatisfactory outcomes [2, 5, 6]. While superficial grade II burns usually heal spontaneously and require minimal intervention, deep grade II burns are associated with prolonged healing and may require surgical intervention [2, 3, 5]. In clinical practice, repeated wound care and application of specialized burn dressings are often used during the first two weeks [1, 2, 5, 7]. Several imaging modalities have been evaluated for their utility in burn wound assessment [4, 6, 8], [9], [10], [11]. Laser Doppler imaging is the most cited, but it lacks the ability to provide structural information of the burn wound [2, 4, 6].

Optical coherence tomography is a technique that uses the reflection of a laser beam to create an image of the epidermis and papillary dermis. Its penetration depth is approximately 2 mm. With dynamic OCT (D-OCT), vascular structure and perfusion can be added to the structural image of the skin [2, 7]. OCT is well-established in dermatology for the evaluation of skin tumors and other skin diseases [12]. Due to its non-invasive nature and high spatial resolution, the technique seems to be well-suited for the diagnosis of thermal injuries [2, 11]. In a pilot study, our research group defined basic epidermal, dermal, and vascular OCT features of thermal injury in children [2]. Damage to the vascular plexus appeared to be particularly relevant. In this study, we examined the depth of the capillary network in unaffected and thermally injured skin. We hypothesized that thermal injury leads to changes in the depth of the capillary network that are prognostically relevant in grade II thermal injury [2].

For this purpose, we determined the depth of the papillary plexus of healthy skin on the anterior body wall, a frequently affected body area in a well-defined age group. We compared these measurements to those of thermal injuries that were classified by clinical judgment in grade IIa and IIb injuries.

## Patients and methods

Our single-institution study was approved by the local ethical committee of the University of Lübeck (10.06.2015, File Number 15-116). Informed consent was obtained from parents and directly from the child when they were older and capable of giving consent.

**Figure 1:**
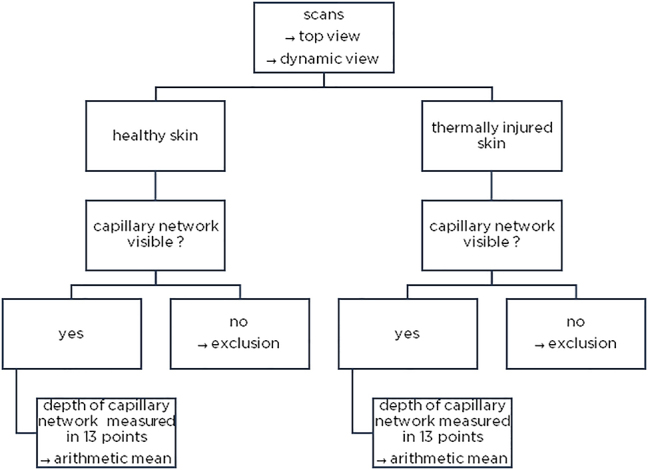
Method illustration – examination of the image material regarding the existence of the capillary network and measurement (top and dynamic view).

Twenty-five children with thermal injuries to the ventral thorax or at adjacent sites, admitted to the Pediatric Burn Center Lübeck between 01/2015 and 12/2019, were included and analyzed for the depth of the capillary network, see Figure 1. All patients received a scan of an injured and uninjured area in acceptable quality.

Demographic information, injury patterns, and wound characteristics were obtained from the electronic medical record.

All children underwent wound care and evaluation 48–72 h after admission as a planned secondary evaluation under general anesthesia.

After dressing removal and disinfection, residual blisters were removed, and photographs were taken. The affected area was then graded as grade IIa or IIb based on clinical judgment: a moist and red wound bed was classified as grade IIa, and dry wounds with a white wound bed and capillary hemorrhage were classified as grade IIb.

An area of unaffected skin adjacent to the wound was selected for the OCT control/reference scan. For the OCT injury scan, the most severely affected area of thermal injury according to our clinical assessment, most often the central area, was selected. The observer was blind to the depth of the papillary plexus, and all measurements and calculations were performed separately.

Burn wounds were dressed with a lactid-trimethylencarbonat-caprolactone copolymer membrane (Suprathel^®^, PolyMedics Innovations GmbH, Denkendorf, Germany).

Clinical outcome data, including scar assessment based on photographs and the Vancouver scar sccale, were prospectively collected in a pseudomized registry. Patients were followed in our outpatient clinic. Children with spontaneous wound healing and no scarring were discharged after wound healing and skin color recovery. Children with deeper wounds and scarring were treated with compression garments when indicated, and scar maturation was monitored in the outpatient clinic.

### Optical coherence tomography

OCT was performed with the Vivosight Dx^©^ Optical Coherence Tomography device (Michelson Diagnostics Ltd., Kent, UK) and the Vivosight OCT Analysis Research tool (version 4.0.99.17^©^).

Each OCT examination consisted of 120 consecutive scans with an area of 6×6 mm and a depth of 2 mm. The images were displayed as consecutive longitudinal sections and as 3D images representing an en-face view. This en-face view, combined with the flow signal from the D-OCT, was necessary to visualize the vessel pattern. Using multi-beam technology, the swept-source-based Fourier OCT provides a resolution of <6 µm axially and <7.5 µm laterally.

### Measurements

**Figure 2:**
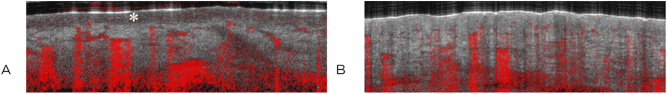
Representative images of healthy skin (A) with epidermis (∗) and thermally injured skin grade IIa (B) in comparison, sagittal view. The red color indicates movement, such as blood flow.

**Figure 3:**
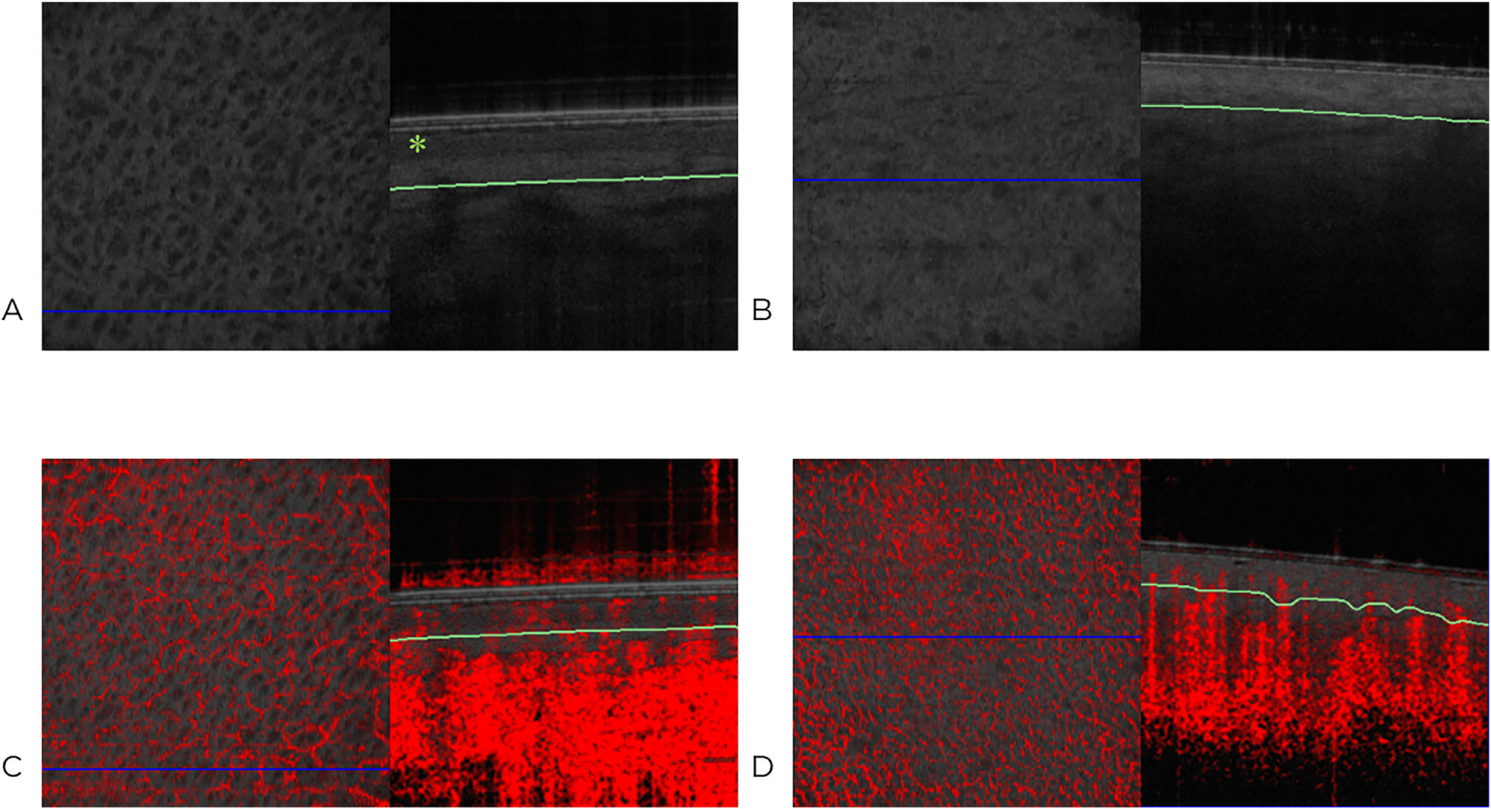
Representative optical coherence tomography images of healthy skin (A) with epidermis (*) and thermally injured skin grade IIa (B) [en-face view (left) and sagittal view (right)]. Dynamic optical coherence tomography image of healthy thoracic skin (C) and thermally injured skin (D) [en-face view (left) and sagittal view of the vascular plexus (right)]. The green line marks the depth of measurement of the plexus, and the blue line marks the location of the sagittal section shown on the right in the scanned area. The red color indicates motion, such as blood flow.

Three groups were created for comparison: 1 – healthy skin (control), 2 – grade IIa thermal injuries, 3 – grade IIb thermal injuries. For each examination, 13 measurements were taken from the surface to the vascular signal and the arithmetic mean was calculated. Examples are shown in Figures 2 and 3.

**Figure 4:**
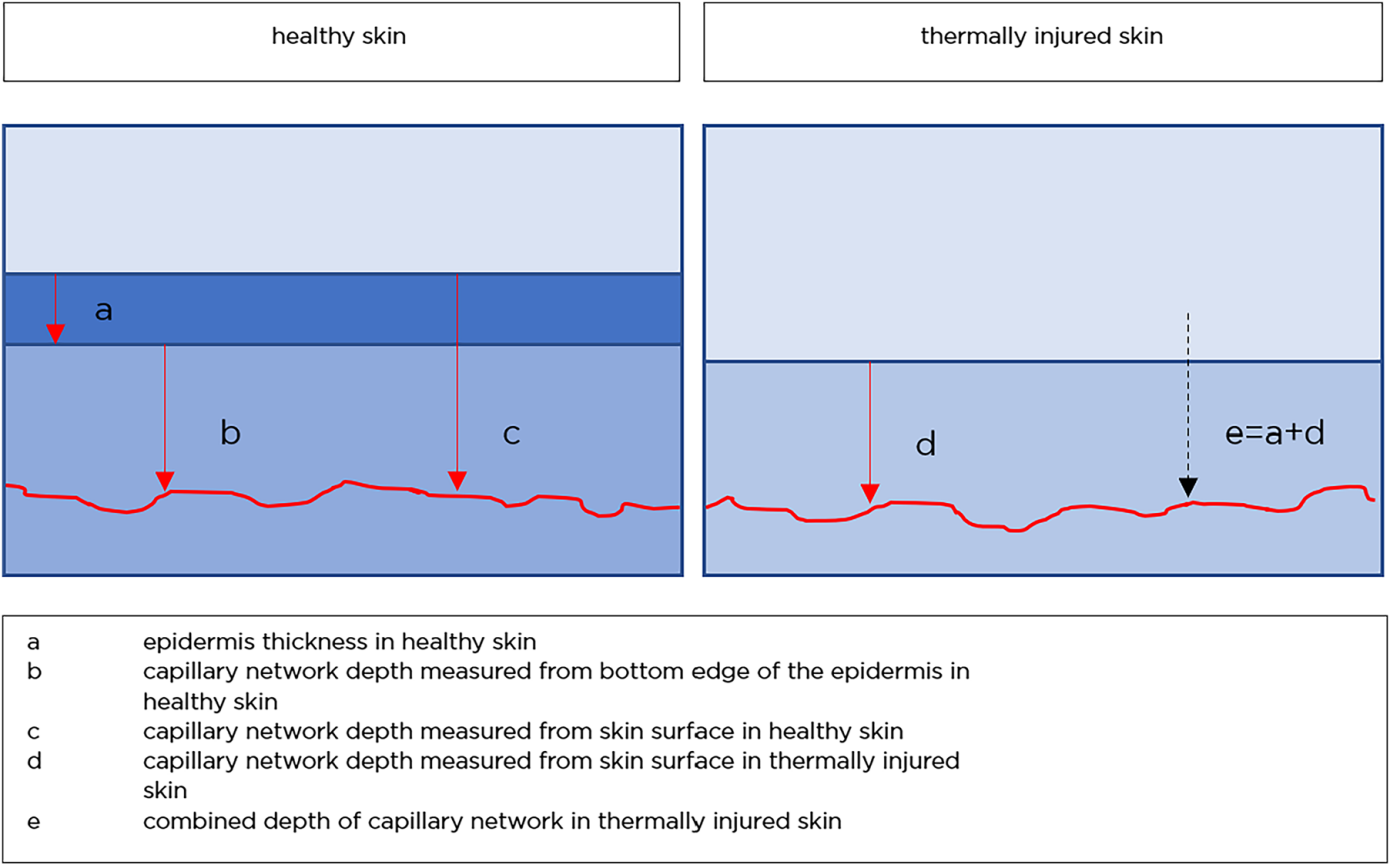
Schematic illustration of skin layers and the combined depth.

By definition, second-degree thermal injuries show destruction of the epidermal layer and blistering within the dermal layer. For better comparison of measurements, a “combined papillary plexus depth” has been defined. This measure combines the depth of the papillary plexus at the injury site with the mean epidermal thickness of healthy skin in the individual patient (Figure 4). This measure was introduced to account for individual variations in epidermal thickness.

### Analysis

Measurements were collected in Microsoft Excel^®^ and, after coding, transferred to SPSS^®^ (version 26) for statistical analysis. Biometric consultation was provided by the Department of Statistics and Biometry at the University of Lübeck.

Group comparisons were performed using Fisher’s exact test and Mann–Whitney U test. The Kolmogorov–Smirnov test was used to test for normal distribution. The standard significance level was α=0.05. The whiskers of the box plots indicate the minimum and maximum values. Outliers are shown as dots, in which case the whiskers indicate the closest minimum and maximum values. ROC curve modeling was used to assess model quality. The correlation between clinical grade and difference in capillary network depth was tested using binary logistic regression analysis.

## Results

From 2015 to 2019, 25 consecutive patients presenting with thermal injuries of the ventral thorax to the Department of Pediatric Surgery at the University of Lübeck were included. Patient characteristics are shown in Table 1. The total body surface area (BSA) affected ranged from 2 to 35 %. The median length of hospital stay was 7 days (2–45 days), with a median of three surgical procedures/bandage changes under sedation or anesthesia (1–11). Wound healing was observed at a median of 12 days.

**Table 1:** Characteristics of patient cohort – children with thermal injuries to the thorax.

Categories	Cases
Sex	Male (n=16)
	Female (n=9)
Age	7 months to 15 years
	Median 18 months
	Mean age 3.51 years, SD 4.41
	<1 year: 16
Etiology	Scald (n=21)
	Burn (n=4)
Clinical depth	Injury grade IIa (n=16)
	Injury grade IIb (n=9)

Follow-up information is available for 21 of the 25 children (84 %; median 14 months, range 1–44 months).

In 19 cases, the outcome was excellent or mildly impaired Vancouver scar scale (VSS) ranging from 0 to 4 with complete restoration of normal skin texture and patient discharge with a maximum follow-up of 29 months.

Children with grade IIa injuries all had a VSS of less than 3 at discharge with a maximum follow-up of 29 months.

Of those with clinical grade IIb injuries, three of nine healed with some persistent scarring. One child with a grade IIb injury with conservative therapy had a maximum VSS of 5, which improved to a VSS of 3 with compression garments. Two patients required a split skin graft. Grafting was performed 12 and 15 and days after injury with standard post-skin graft care and showed an excellent result with an elastic scar VSS 3 and 1 (Vancouver Scar Scale) 13 and 44 months after injury.

In Figure 2, the epithelium (*) is showed up as a dark band (Figure 2A), which is not visible in the injured skin (Figure 2B); subsequently, the capillary plexus moves closer to the surface.

In Figure 3, the epithelium (*) can be visualized as a dark band in healthy skin (Figure 3A), which cannot be shown in thermally injured skin (Figure 3B). In D-OCT, the capillary network is visible (Figure 3C and D).

Figure 4 shows how the measurements were taken. The concept of the “combined depth” is illustrated. The red line is indicating the papillary plexus.

### Epidermal thickness and depth of capillary network in healthy skin

**Figure 5:**
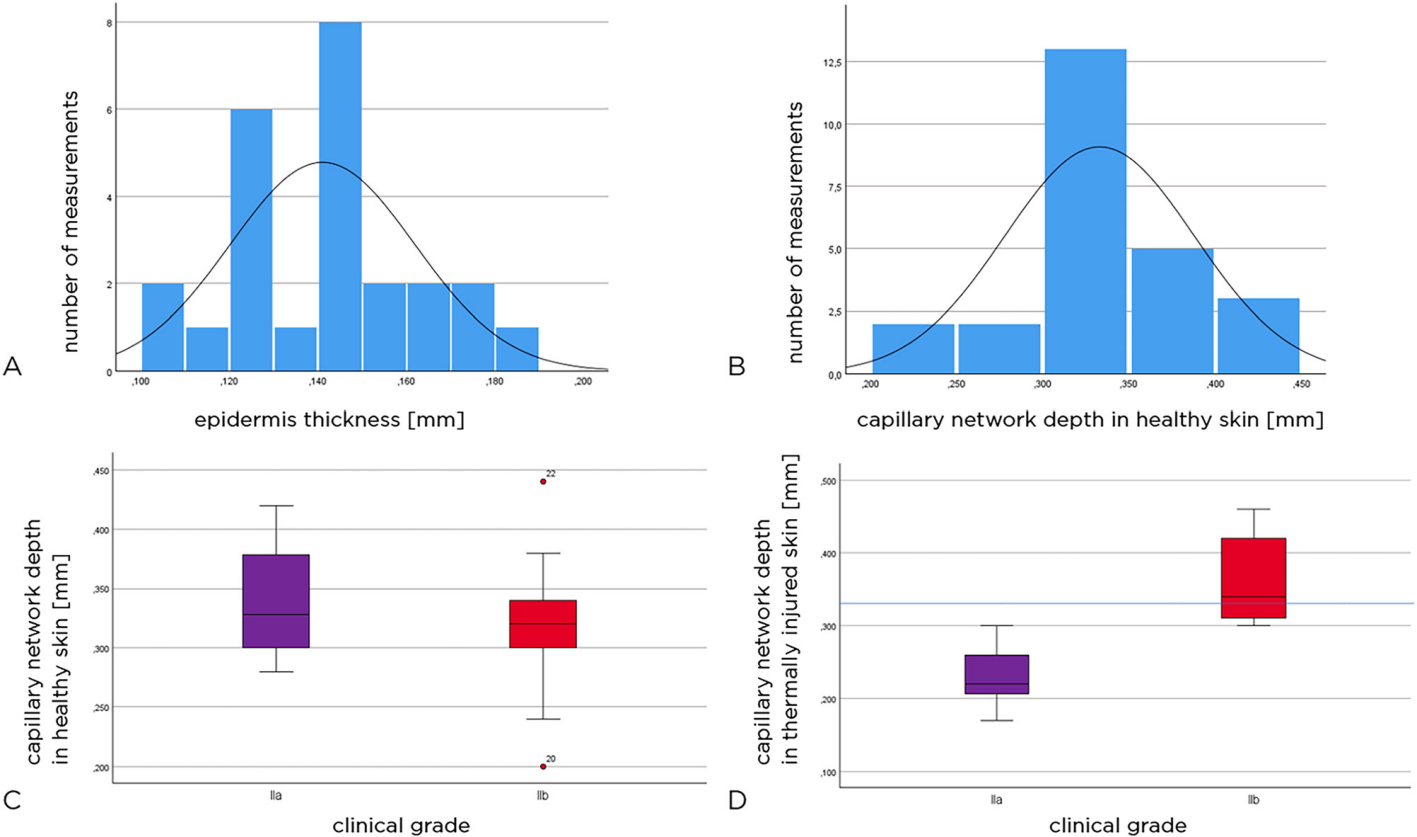
Epidermal thickness (mm), all patients (A). Papillary plexus (all patients) (B). Height of the capillary network (mm) in the healthy skin adjacent to the injured area (C). Papillary plexus height (mm) of the injured area. The blue line shows the average level of the capillary network in the healthy skin of the entire cohort of patients (D).

The epidermal thickness of healthy skin and the depth of the papillary plexus could be measured in all 25 children. The mean value of epidermal thickness in healthy skin was 0.14 mm (SD±0.02 mm), and the values were normally distributed (in Figure 5A) (Kolmogorov–Smirnov test: p=0.200). Papillary plexus depth values in healthy skin also showed a normal distribution (Figure 5B).

### Depth of capillary network in healthy skin

In patients with grade IIa thermal injuries, the depth of the capillary network in healthy skin averaged 0.34 mm (SD±0.06 mm) and in patients with grade IIb wounds 0.32 mm (SD±0.07 mm).

The box plots in Figure 5C show the depth of the capillary network in healthy skin. There was no statistically significant difference between the groups (Mann–Whitney U test: p=0.669).

### Depth of the capillary network in thermally injured skin

16 children had grade IIa thermal injuries and nine had grade IIb thermal injuries. The depth of the papillary plexus in the thermally injured skin was found to be 0.28 mm (mean, SD±0.079 mm), with 0.23 mm (SD±0.04 mm) in the grade IIa injury group and 0.36 mm (SD±0.06 mm) in the grade IIb injury group. Thus, the capillary network was found to be on average 0.13 mm deeper in the thermally injured skin of the grade IIb group than in the grade IIa group. We note that the two patients requiring skin transplantations had the deepest capillary network visualized at 0.44 and 0.46 mm depth.

The box plots in Figure 5D show the depth of the capillary network in the thermally injured skin in the two groups of patients. There is a significant difference between grade IIa and IIb injuries (Mann–Whitney U test: p<0.001). The blue line indicates the depth of the papillary plexus (mean, all patients).

**Figure 6:**
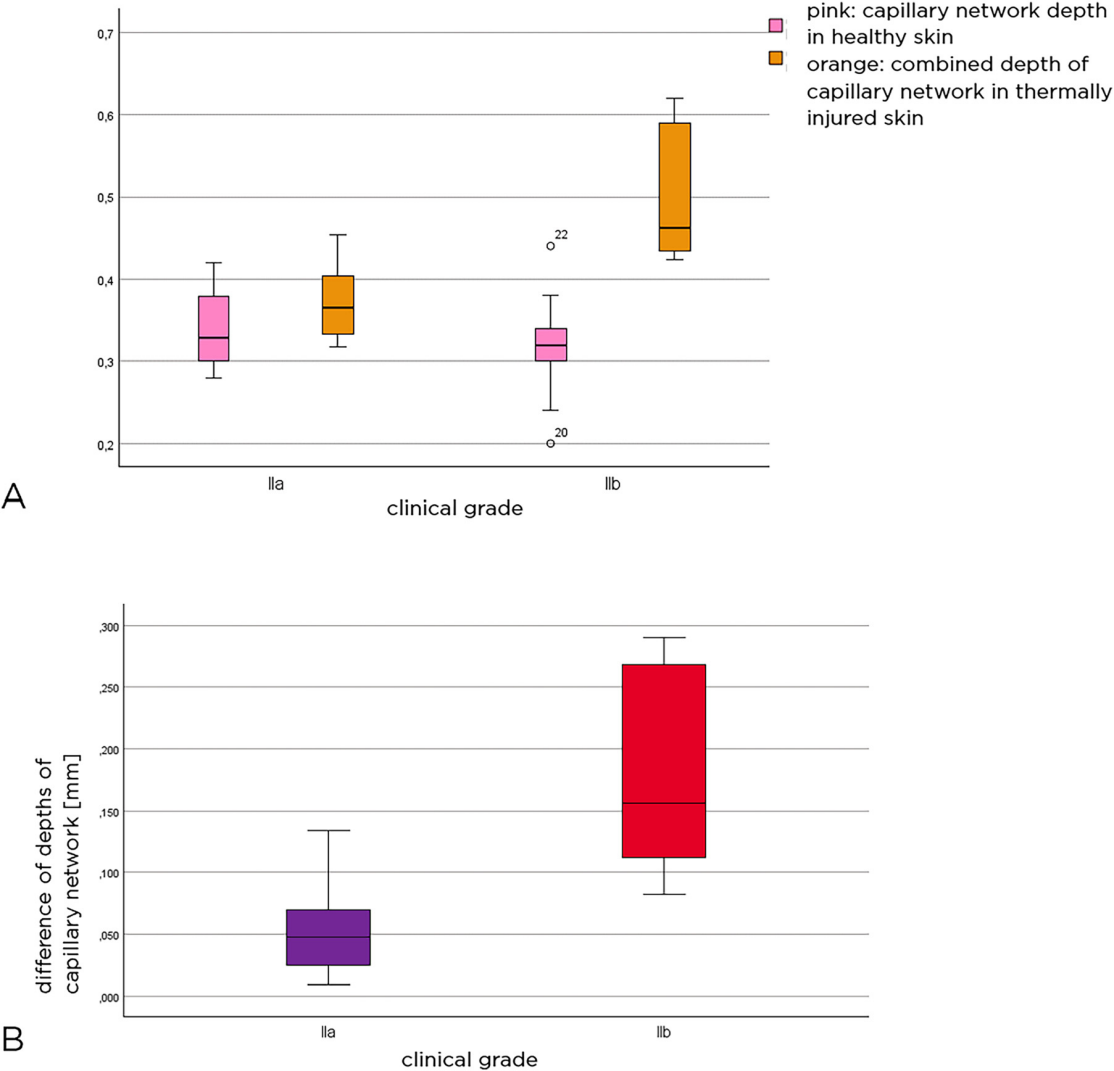
Level of capillary network depth in both healthy and thermally injured skin in relation to patient collectives (A). Difference between the combined depth of the plexus in injured skin and the depth of the plexus in healthy skin (B). Pink color represents the depth of the capillary network in healthy skin, orange color shows the combined depth of the capillary network in thermally injured skin, purple represents the group with grade IIa injuries, while red represents those with grade IIb injuries.

The box plots in Figure 6A provide an overview of the depth of the capillary network in both healthy and thermally damaged skin in relation to the patient collectives. The depth of the capillary network in healthy skin was compared to the combined depth of the capillary network in the injured skin. It was found that the median depth of the capillary network in healthy skin was similar in both patient groups, whereas the median combined depth of the capillary network in thermally injured skin was significantly deeper at 0.46 mm in the patient group with grade IIb injuries than in those with grade IIa injuries.

The difference is not related to the added epidermal thickness, but to the actual depth of the capillary network. Considering the difference between the actual capillary network depth in healthy skin and the combined capillary network depth/capillary network depth in injured skin, the difference in capillary network depth is obtained.

The box plots in Figure 6B show the difference in capillary network depth between healthy and thermally injured skin in the two groups of thermal injuries. It was 0.18 mm (SD±0.08 mm) for grade IIb and 0.06 mm (SD±0.04 mm) for grade IIa. This was statistically significant (Mann–Whitney U test: p<0.001).

**Figure 7:**
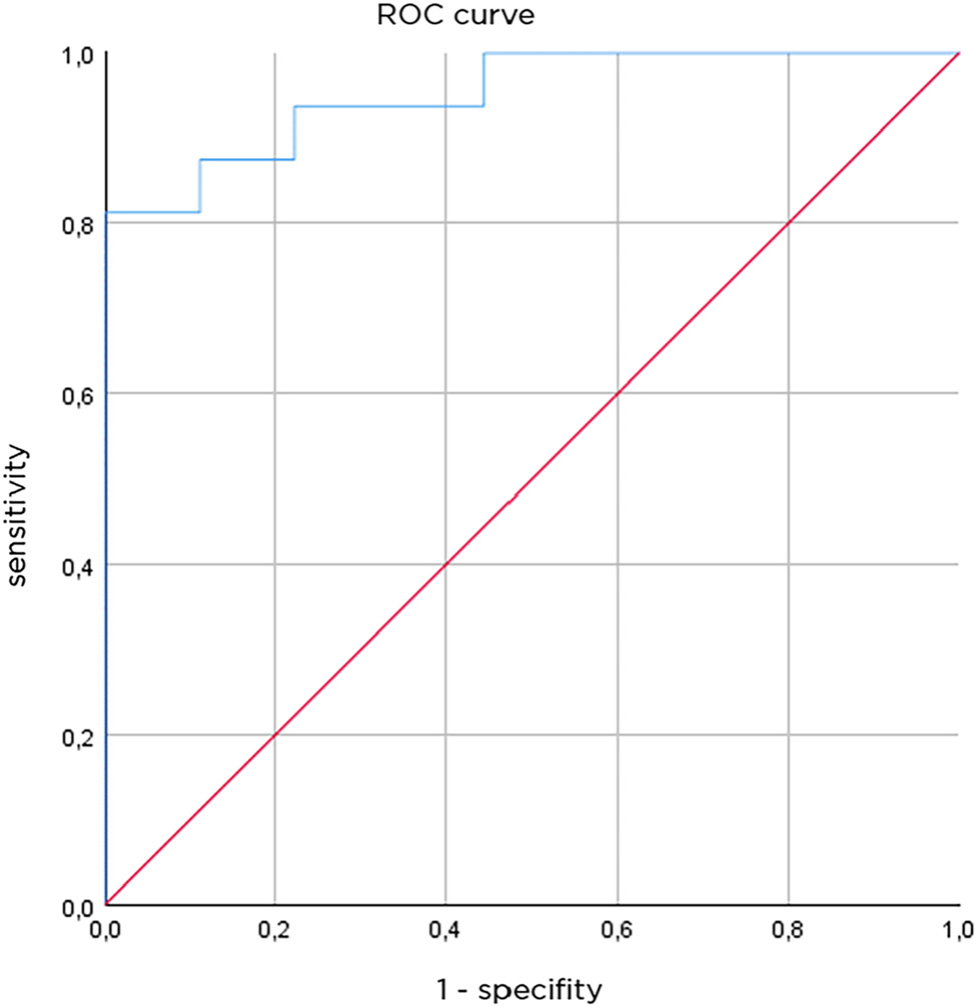
Receiver operating characteristics curve, AUC is 0.951 (n=25 measurement).

To explore the diagnostic value of these measurements in terms of sensitivity and specificity, a receiver operator curve can be constructed. The differences in combined capillary network depth/capillary network depth in thermally injured skin compared to healthy skin are visualized in a ROC curve (Figure 7).

With a separation value of 107.5 µm, a sensitivity of 87.5 % and a specificity of 88.9 %, the results show that the OCT-assigned injury grade/depth reflects the observed clinical grade.

To evaluate the strength of the association between the clinical grade of thermal injury and the difference in capillary network depth, a binary logistic regression analysis model was applied.

The model predicted grade IIa in 14 of 16 cases (87.5 %) in patients who had grade IIa thermal injury at longitudinal follow-up. In seven of nine cases (77.8 %), the model correctly predicted grade IIb in patients with grade IIb thermal injury. This corresponds to a sensitivity of 77.8 % and a specificity of 87.5 %. Overall, this method correctly classified 21 of the 25 patients according to the clinically assessed degree of injury, for an overall predictive accuracy of 84 %.

The only cases of disagreement between clinical grading and OCT grading were wounds clinically assigned grade IIb and grade IIa on OCT. These wounds healed spontaneously with good results and maybe classified grade IIa.

The deepest level of the capillary plexus and the greatest calculated difference characterized the two cases requiring skin grafting.

## Discussion

A well-defined study population is needed to assess the value of a new diagnostic technique.

Our patients showed the pattern of injury most commonly reported in the German burn registry [13, 14]. The ventral thorax is the most affected anatomical region, and mixed grade II scalds in young children are the most common lesions [13].

The purpose of this study was to evaluate the diagnostic value of OCT-based measurements of the papillary plexus in thermal injuries. The vascular supply of the burn wound is essential for its ability to heal – an inadequate vascular network results in wound debridement and skin grafting [15–17]. Difficulties in clinical assessment of burn wounds have prompted the search for new imaging technologies [2, 4], [5], [6], [7], [8].

The specific technical advantage of OCT and D-OCT is that blood flow and structural abnormalities of the wound are captured simultaneously and can thus be correlated for diagnostic purposes [2].

Our results indicate that the level of the papillary plexus, as determined by OCT-based measurements, is related to the clinical assessment of grade IIa and IIb thermal injuries.

In grade IIa lesions, blistering and loss of epidermis result in a moist and red wound, with the papillary plexus closer to the surface.

In grade IIb lesions, the upper layers of the plexus are destroyed, deep vessels are sometimes seen with fewer ramifications and interruptions [2]. This is associated with a dry and white burn wound appearance.

In our measurements, the papillary plexus was significantly deeper in grade IIb wounds than in grade IIa wounds and in healthy skin. Therefore, detection of blood flow at a depth of 0.04 mm in such a lesion may indicate a poorer healing prognosis.

Our study also provides new, accurate data on healthy skin anatomy and vasculature. Individual measurements showed minimal variation. Such information may be useful in future studies.

Laser Doppler imaging (LDI) has been reported to improve the diagnostic accuracy of burn wound assessment in a similar clinical setting [6, 18]. The advantage of this technique is that the entire burn area can be examined. However, this modality relies on the detection of blood flow alone – variations in skin temperature and body shape can affect LDI measurements in relevant ways [19, 20].

Laser speckle imaging (LSI) has recently been compared to LDI, and both techniques have proven valuable in predicting the healing potential of thermal lesions [19, 21]. Both techniques provide a macroscopic view of blood flow in the area of interest, but do not detect structural changes in the skin.

The strength of OCT is the high resolution of anatomical details related to blood flow and vascular pattern [2]. Like the other techniques, it is non-invasive, but OCT is the closest technique to an *in vivo* biopsy that also visualizes the structural features as well. The weakness of OCT is the small area that can be visualized in one measurement. As with a biopsy, OCT carries the risk of “sampling error” if an unrepresentative area of the wound is examined. Like any other available technique LDI and LSI, OCT cannot penetrate burn wound dressings.

In summary, OCT provides high-resolution images of burn wounds that allow highly accurate measurement of the depth of the papillary plexus. In this study, significant relations between burn depth and depth of the papillary plexus were found.

OCT is rapidly evolving. It can assess the effect of topical treatments and skin debridement in real time. Despite its limitations, OCT is a valuable diagnostic technique that should be further developed for assessment of burn wounds.
